# Cosmeceutical Peptides in the Framework of Sustainable Wellness Economy

**DOI:** 10.3389/fchem.2020.572923

**Published:** 2020-10-30

**Authors:** Fosca Errante, Patrycja Ledwoń, Rafal Latajka, Paolo Rovero, Anna Maria Papini

**Affiliations:** ^1^Laboratory of Peptide and Protein Chemistry and Biology, Department of Neurosciences, Psychology, Drug Research and Child Health-Section of Pharmaceutical Sciences and Nutraceutics, University of Florence, Firenze, Italy; ^2^Espikem S.r.l., Prato, Italy; ^3^Department of Bioorganic Chemistry, Faculty of Chemistry, Wroclaw University of Science and Technology, Wroclaw, Poland; ^4^Laboratory of Peptide and Protein Chemistry and Biology, Department of Chemistry “Ugo Schiff”, University of Florence, Firenze, Italy; ^5^PeptLab@UCP and CY Cergy Paris Université, CNRS, BioCIS, Cergy Pontoise, France

**Keywords:** signal peptides, neurotransmitter peptide inhibitors, carrier peptides, sustainable wellness economy, peptide-based cosmetics

## Abstract

Among the many aspects that contribute to the wellness of each individual, healthy and younger-looking skin play a relevant role, as clearly shown by the important growth of the skin-care products market observed in recent years. In this scenario, the field of cosmeceuticals appears particularly promising, being based on cosmetic products containing active ingredients. Among these, several peptides were proposed for cosmeceutical applications, thanks to their specific interaction with biological targets. In this mini-review, we report some of the most investigated and used peptides for cosmetic formulations, taking into account that cosmeceutical peptides are basically divided into three main categories (i.e., neurotransmitter inhibitors, carriers, and signal peptides). Special attention was payed to the scientific studies supporting the claimed biological activity of these peptides, as a fundamental aspect that should underpin the growth of this field in the framework of a sustainable wellness economy.

## Introduction

In 2015 the United Nation defined 17 Sustainable Development Goals (SDG) that can contribute to change the world for the better. The third of these goals is to establish “good health and well-being”, thus indicating that health and wellness are strictly linked. Among the many aspects that contribute to the wellness of each individual, healthy and younger-looking skin play a relevant role, as clearly shown by the important growth of the skin-care products market observed in recent years (Global Wellness Institute, 2018). This is a wide market segment, ranging from pure cosmetics to dermatological products and including the so-called cosmeceutical products, which lie in a gray area between cosmetics and drugs and, despite their large use, to date are not recognized from the Food and Drug Administration.

Peptides entered the cosmeceutical field in 1973 when Pickart proposed the synthetic peptide GHK as a signal peptide enhancing collagen production and acting as a carrier peptide when complexed with Cu(II) (Pickart and Thaler, 1973). Since then, thanks to their versatility, a plethora of peptides of cosmeceutical interest have been developed in response to the most frequent and not fully satisfied market requests.

Perception of beauty is tied to the idea of youth. This is why a large number of peptides proposed in the field of cosmetics are used as anti-aging products. In this field several products to adorn skin have grown in popularity and they are sought after by women but also men. External agents, such as smog, together with endogenous pathways, such as oxidative stress, affect skin cell oxidative damages, resulting in skin aging. To adjust the skin biological clock, it is necessary to get and maintain a good balance between structural protein synthesis and degradation. In fact, most of the products on the market act *via* this mechanism, regulating collagen turnover. A second possible pathway is to obtain a temporary improvement of skin firmness by blocking or promoting some neurotransmitters, thus leading to a decrease of fine and age-induced wrinkles. Care should be taken to the association between good topical care products and food supplements in which the term “peptide” is starting to be (ab)used to identify mostly not well identified hydrolysates. Nevertheless, women today understood the importance of reading product labels and INCI (International Nomenclature of Cosmetic Ingredients) lists. In fact, more and more women ask for specific information regarding the active principles used in cosmeceuticals and they are attracted by scientific data demonstrating the number of claims reported by the cosmetic industry. It has to be clearly emphasized that cosmeceuticals, differently from cosmetics, contain active ingredients whose activity claim should be supported by scientific investigations. Only at these conditions the use of active ingredients in cosmetic formula is acceptable (Papini, 2010).

**Figure 1:**
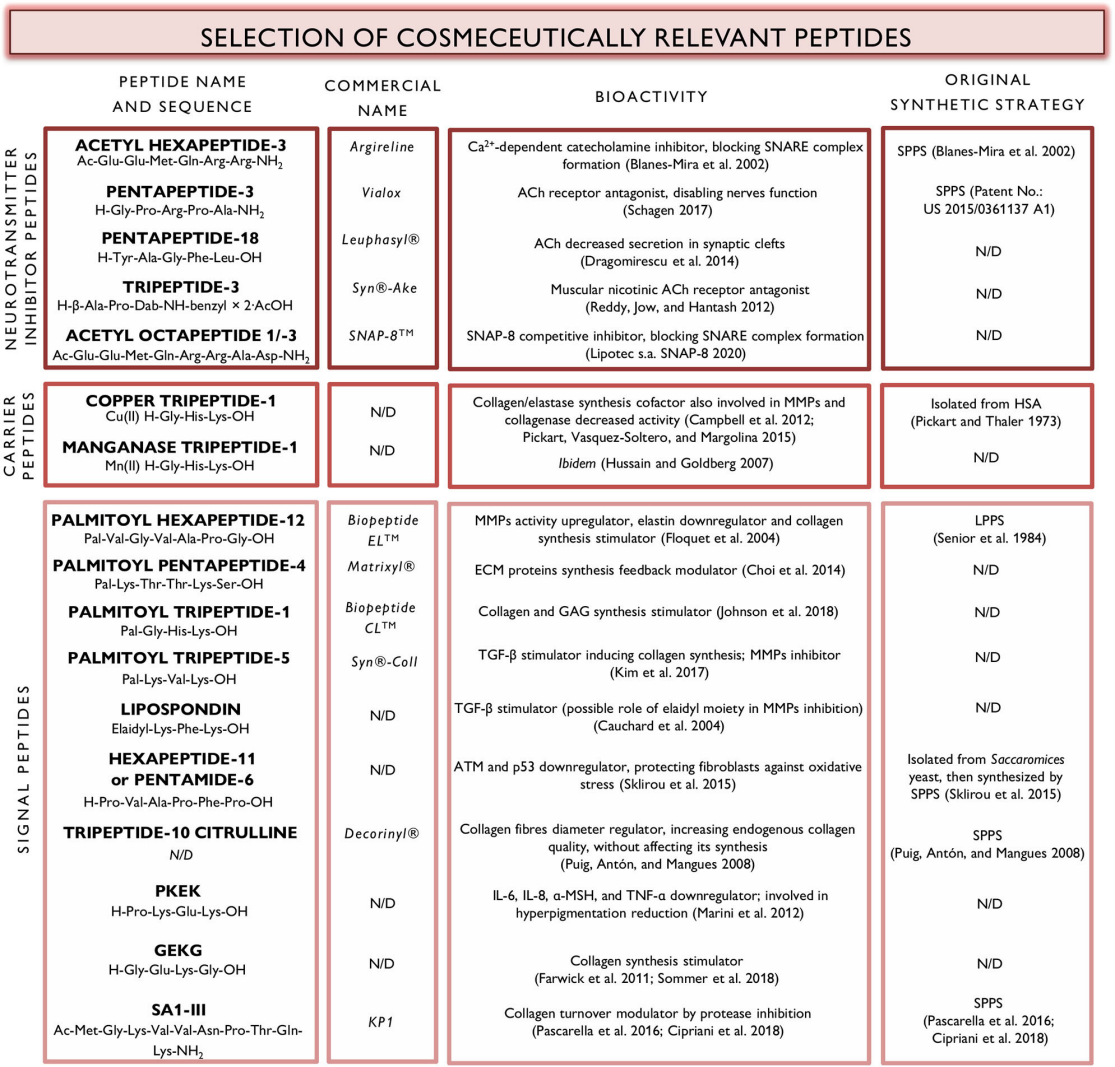
Relevant peptides in commercially available cosmeceuticals.

In this mini-review we will list some of the newest synthetic peptide-based cosmeceuticals discovered in the last decade, classified as neurotransmitter inhibitor peptides, carrier peptides and signal peptides. Figure 1 also reports their bioactivity and when available, original synthetic strategy.

## Neurotransmitter Inhibitor Peptides

Fine lines and wrinkles are getting formed, among other, due to the muscle cramps. These movements, provoked both involuntary and knowingly, are strictly connected with large number of SNARE (*Soluble N-ethylmaleimide-sensitive factor Activating protein REceptor)* complexes. Acetylcholine (ACh), the main neurotransmitter involved in this process, is released from vesicles as the result of a reaction cascade mediated by SNAP (*SyNaptosome-Associated Protein*) receptor protein. It controls directly the synaptic vesicle fusion for ACh release, involving SNARE complex formation (Lima and Pedriali Moraes, 2018). Once ACh is released, it binds to the appropriate receptor and triggers muscle cramps. Some peptides with similar sequence to the synaptic proteins can potentially inhibit this reaction. Therefore, those are termed *neurotransmitter inhibitor peptides* (Schagen, 2017).

SNAP-25 (*SyNaptosome-Associated Protein*, molecular weight 25 kDa), which is essential for ACh release from vesicles with the presynaptic vesicle, is particularly targeted by the well-known botulinum neurotoxin type A. Botulinum neurotoxin type B has a different mechanism of action. In fact, it leads to VAMP (*Vesicle-Associated Membrane Protein*) cleavage, necessary for the release of ACh (Lupo and Cole, 2007). Botulinum toxins are widely applied as a reference while neurotransmitter inhibitor peptides are tested as cosmeceuticals.

### Acetyl Hexapeptide-3 (*Argireline*)

One of the best known peptide with neurotransmitter-inhibiting properties is a synthetic hexapeptide, so-called acetyl hexapeptide-3 and coined *Argireline*. The sequence Ac-Glu-Glu-Met-Gln-Arg-Arg-NH_2_ (Blanes-Mira et al., 2002), covers the N-terminal fragment domain of SNAP-25, promoting the inhibition of Ca^2+^-dependent catecholamine release through the interrupted formation of SNARE complex. In comparison to the botulinum toxin, Argireline presents similar potency but lower efficacy. However, it can be considered as a safe, non-toxic alternative for botulinum family compounds.

Recent data on acetyl hexapeptide-3, reported in the literature, comprise its skin permeability, also with the use of microneedles (Krishnan et al., 2014; Zhang et al., 2014). Skin permeation depends on various factors (e.g., pH, charge, molecular weight, concentration, and background electrolytes). These characteristics will have always to be taken into consideration during peptide delivery optimization. Microneedles enhance this process even 40-fold compared to passive diffusion. The clinical studies with Argireline peptide confirmed its efficiency and its potential as an active ingredient to be used in cosmeceuticals (Tadini et al., 2015; Raikou et al., 2017). Very recently, Lim et al. reported a relevant, wide study on acetyl hexapeptide-3, designing eye patches with microneedles to increase peptide delivery (Lim et al., 2020). Skin permeation was successfully improved also by nanoliposome delivery, resulting with significantly more elastic skin and diminished wrinkles (Han et al., 2020).

### Pentapeptide-3 (*Vialox*)

*Vialox* is a pentapeptide derived from snake venom (H-Gly-Pro-Arg-Pro-Ala-NH_2_) that was demonstrated to induce muscle relaxation. It acts as an antagonist of ACh receptor, disabling the function of nerves and preventing unwanted cramps (Schagen, 2017). Up to date, there are no further investigations directly describing or enhancing anti-wrinkle properties of Vialox. The only available data are provided by the manufacturer, which reports that *in vitro* and *in vivo* studies warrant Vialox efficacy because of wrinkle size and skin roughness decrease.

### Pentapeptide-18 (*Leuphasyl*)

The pentapeptide-18 composed of the sequence H-Tyr-Ala-Gly-Phe-Leu-OH, was reported for the first time in 2015. It became one of the most popular active peptide ingredients in anti-aging formulations (Lipotec s.a., 2005). It acts like enkephalins, decreasing the ACh secretion in the synaptic cleft. Both instrumental and volunteer-involving studies acknowledge its effectiveness in wrinkles reduction (Dragomirescu et al., 2014). It is worth to notice, that a synergistic effect of Leuphasyl and Argireline has been proved (Lipotec s.a., 2005).

A very recent study, carried out by Park et al. and published in 2020, concerns D-tyrosine-containing cosmetic peptides. Their role in melanogenesis process was clearly demonstrated (Park et al., 2020). In particular synthetic Leuphasyl-derived peptides, obtained replacing N-terminal L-tyrosine with D-tyrosine or adding L/D-tyrosine at the C-terminus, were tested on human melanoma MNT-1 cells. The decrease of melanogenesis, particularly in the case of Leuphasyl peptide analogs containing C-terminal D-tyrosine, was confirmed, leading to a remarkable cosmeceutical with dual-anti-wrinkle and whitening properties.

### Tripeptide-3 (*Syn-Ake*)

Another relevant example of peptide, acting similarly to the aforementioned Leuphasyl, is the Tripeptide-3 (H-β-Ala-Pro-Dab-NH-benzyl × 2·AcOH) (e.g., Dipeptide Diaminobutyroyl Benzylamide Diacetate), commercially known as *Syn-Ake* (Reddy et al., 2012). As a reversible antagonist of muscular nicotinic ACh receptors, Tripeptide-3 prevents ACh binding to the receptor. Afterwards, sodium ion channels remain closed, depolarization does not take place, and muscles cannot cramp. The manufacturer claims in the information, *in vitro* and *in vivo* studies about the reversible mechanism of action (0.5 mM concentration), but put also in evidence that 28-days application of *Syn-Ake*, reduced the visibility of wrinkles up to 52% (DSM, 2020).

To the best of our knowledge, until now there are no scientific papers describing clinical studies or modifications of Tripeptide-3. However, different formulations containing this peptide are available on the market: face creams, serums, and eye patches (DSM, 2020).

### Acetyl Octapeptide-1/-3 (*SNAP-8*)

One the latest discovery of cosmeceutical industry includes Acetyl Glutamyl Heptapeptide-3 (Ac-Glu-Glu-Met-Gln-Arg-Arg-Ala-Asp-NH_2_) and SNAP-8 (e.g., Acetyl Octapeptide-1 or-3) (Lipotec s.a., 2020). Muscle contraction is decreased, due to the modulated formation of SNARE complex, in which SNAP-8 is mimicking SNAP-25 and makes it acting as a competitive inhibitor. Consequently, glutamate release is declined and inhibition level spans 43% in the case of 1.5 mM concentration of SNAP-8. According to the data available in the manufacturer's website, maximum wrinkle reduction strives for−62%, with the mean value at the level of −35%. It is also worth to be remarked that *in vitro* assay highlighted an independent mechanism but synergistic effect between SNAP-8 and *Leuphasyl*, each one tested independently and resulting with 38% and 7% inhibition, respectively. In the trial, including both of them in equal concentrations (0.75 mM), a total inhibition value of 47% was observed.

## Carrier Peptides

Cu(II), one of the crucial metal ions in human body, can be stabilized or delivered into cells among others, by peptides. It is incorporated in various processes, including enzymatic reactions, wound healing, and angiogenesis (Husein el Hadmed and Castillo, 2016). In the case of cosmeceuticals, copper is an essential cofactor for lysyl oxidase over the formation of collagen or elastin. Reducing the activity of MMPs (Matrix metalloproteases) and collagenase, prevent premature skin-aging (Blanes-Mira et al., 2002; Lupo and Cole, 2007). The most important peptide of this class is Gly-His-Lys.

### Copper Tripeptide-1 (Cu-GHK)

H-Gly-His-Lys-OH, isolated for the first time from human serum albumin (HSA) and proposed also as palmitoylated derivative, was demonstrated to be able to complex Cu(II) spontaneously, thus simplifying its absorption (Pickart and Thaler, 1973; Pickart and Margolina, 2018). Different scientific papers confirmed skin refining after the application of GHK, entering also into details of its mechanism of action (Campbell et al., 2012; Pickart et al., 2015).

Two main modifications of Cu-GHK peptide include: Pal-GHK with a palmitoyl moiety linked at the N-terminus (as discussed in Signal peptides section) and a biotin-complex derivative, both designed to improve physical stability and transdermal delivery (Arul et al., 2005; Jeong et al., 2019).

The most recent studies involving Cu(II)-tripeptide (e.g., Cu-GHK) refer to oligoarginine conjugation (Hur et al., 2020) and D-amino acid modifications, as in the case of *Leuphasyl* (Lipotec s.a., 2005). GHK-R4 (a peptide with four arginine residues at the C-terminus), has been confirmed to exhibit dual activity: MMP inhibitory effect and UVB exposure skin protection. Considering its cell penetration properties, it can be suggested that oligoarginine analogs are excellent candidates for cosmeceutical formulations. On the other hand, a C-terminal D-tyrosine in the GHK sequence provides additional whitening properties, decreasing melanin production.

### Manganese Tripeptide-1 (Mn-GHK)

Hussain et al. investigated the influence of manganese complex with GHK peptide (Hussain and Goldberg, 2007). Their study, based on twice a day application for two weeks, showed that Mn-GHK can be a potent ingredient in cosmeceutical formulations, in comparison with Cu-GHK, acting mainly on wrinkles and reducing skin hyperpigmentation.

## Signal Peptides

In this class of cosmeceuticals, those peptides able to modulate skin protein turnover are included, most of them enhancing collagen production. In fact, their name derives from the ability to signal or mimicking the signal occurring in the synthesis of extracellular matrix (ECM) proteins. In this category, peptides promoting collagen production and released by the ECM, are called also matricins (Schagen, 2017). One of the first peptide used with a similar activity is the gastrin-releasing peptide, a bombesin-like neuropeptide that promotes wound healing by stimulating keratinocyte proliferation (Yamaguchi et al., 2002). We will report several other recently discovered signal peptides, which are among the most used in cosmeceuticals.

### Palmitoyl Hexapeptide-12 (*Biopeptide EL™*)

*Biopeptide EL*™ is an analog of the peptide sequence H-Val-Gly-Val-Ala-Pro-Gly-OH, a hexapeptide that was discovered working on elastin derived peptides (Senior et al., 1984). In fact, this peptide sequence is repeated several times in human and other animal elastins. It stimulates collagen production and decreases the synthesis of elastin, with chemotactic activity and metalloproteinases upregulation properties (Floquet et al., 2004). The addition of the palmitoyl moiety at the N-terminus confers enhanced permeation ability, and the resulting peptide is used in a wide range of cosmeceuticals. Sederma, a group from Croda International company, proposes *Biopeptide EL*™ that is a mixture of Palmitoyl hexapeptide-12 with glyceryl polymethacrylate and PEG-8. The mixture is claimed for anti-aging properties thanks to its ability to stimulate fibroblasts mobility (Croda International Plc, 2020a).

### Palmitoyl Pentapetide-4 (*Matrixyl®*)

*Matrixyl*® is the new version of Pentapeptide-4 (palmitoylated Lys-Thr-Thr-Lys-Ser-OH), the well-known signal peptide fragment of the C-terminal propeptide of type I collagen. It stimulates feedback regulation of new collagen synthesis and ECM proteins. The conjugation with the palmitoyl moiety results in more effective delivery across the skin and better stability to skin proteases (Choi et al., 2014).

*In vivo* studies on 93 women, including placebo subjects, and conducted in a double-blind clinical study, show that an application of that palmitoylated peptide improves reduction of fine lines and wrinkles (Robinson et al., 2005). An advanced study on new analogs of Pentapetide-3 was recently published (Tałałaj et al., 2019).

### Palmitoyl Tripeptide-1 (*Biopeptide CL™*)

As above described, Tripeptide-1 (H-Gly-His-Lys-OH) is a carrier when in complex with Cu(II). In reverse, the peptide itself is a signal peptide able to enhance collagen production. *In vitro* and *in vivo* studies showed an increased stimulation of collagen and glycosaminoglycan (GAG) synthesis with a visible reduction of wrinkles in terms of length, depth, and roughness. Palmitoyl Tripeptide-1 is used in *Biopeptide CL*™ and in combination with Pal-GQPR in *Matrixyl*™ *3000*, both developed by Sederma as an anti-aging serum, stimulating collagen production (Cosmetic Ingredient Review, 2012; Johnson et al., 2018; Croda International Plc, 2020b). Its activity is comparable to retinoids, but *Biopeptide CL*™ has the big advantage not to induce skin irritation.

### Palmitoyl Tripeptide-5 (*Syn®-Coll*)

Palmitoyl Tripeptide-5 is composed only of lysine and valine residues (H-Lys-Val-Lys-OH) with a palmitoyl moiety at the N-terminus. This patented peptide is commercialized with the brand *Syn*®*-Coll* by DSM. It acts stimulating TGF-β, a growth factor, which induces collagen biosynthesis, and inhibits matrix metalloproteases degrading collagen. The visible effects of this mechanism of action are improved firmness and elasticity of the skin.

It is also described that conjugation of an L-ascorbate moiety (AA) at the C-terminus (Pal-KVK-AA) is beneficial because it inhibits melanin synthesis *in vitro*, which is translated into a depigmentation effect. *In vivo* data are also available in the literature (Kim et al., 2017).

### Lipospondin

Lipospondin is the elaidyl conjugate of the peptide sequence KFK (Elaidyl-Lys-Phe-Lys-OH). The fatty acid moiety is possibly inhibiting MMPs mRNA and the tripeptide itself stimulates TGF-β. It is also demonstrated that the peptide upregulates collagen and tissue MMP-1 inhibitors production (TIMP-1), and downregulates MMP-1 in fibroblasts (Cauchard et al., 2004).

### Hexapeptide-11 or Pentamide-6

This double-named peptide (H-Pro-Val-Ala-Pro-Phe-Pro-OH) was isolated for the first time from *Saccaromyces* yeast fermentation. It was reported to protect fibroblasts against oxidative stress-mediated premature cellular senescence by mediating a downregulation of cellular proteins, such as ataxia telangiectasia mutated (ATM) and p53, which are hyperactive in degenerative aging pathologies. *In vivo* data also showed an improvement in skin elasticity (Sklirou et al., 2015).

### Tripeptide-10 Citrulline (*Decorinyl®*)

Tripeptide-10 citrulline, named also T10-C, includes a citrulline residue in the sequence of Tripeptide-10, and it is commercialized by Lipotec as *Decorinyl*®. It regulates collagen fibers diameter, increasing the quality of the endogenous collagen, without affecting its synthesis (Puig et al., 2008). It is structurally related to decorin, a human protein influencing fibrillogenesis, and interacts with several proteins or factors involved in various physiological cascades. Due to chronologically skin aging, decorin concentration decreases more and more and a use of T10-C peptide could mimic this function.

Raikou et al. also tested its activity in combination with the well-known acetyl hexapeptide-3, demonstrating that a formulation composed of both of them could provide synergistic rejuvenation effects to the skin (Raikou et al., 2017).

### PKEK

The tetrapeptide H-Pro-Lys-Glu-Lys-OH is used in different formulations for skin depigmentation. In particular, this peptide was reported to reduce pigmentation by decreasing interleukin-6 and -8 (IL-6 and IL-8), α-melanocyte-stimulating hormone (α-MSH), and tumor necrosis factor-α (TNF-α) expression in *in vitro* and double-blind clinical studies (Marini et al., 2012).

Successful delivery of the hydrophilic and polar peptide sequence, was achieved with nano-sized carrier systems, which improved topical bioavailability (Neubert et al., 2018).

### GEKG

The tetrapeptide H-Gly-Glu-Lys-Gly-OH is a collagen synthesis stimulator, developed by an *in silico* molecular design. It is shown that, in comparison with placebo, this tetrapeptide significantly decreases skin roughness, and thereby wrinkles (Farwick et al., 2011; Sommer et al., 2018).

A recent study showed that conjugation with vitamin C by a succinyl moiety as a linker [e.g., ascorbyl succinyl tetrapeptide (AST)], increases expression of basement proteins resulting in a synergistic anti-aging effect (Jeong et al., 2019).

### SA1-III (*KP1*)

As last example, we report a peptide, recently described by our group (Pascarella et al., 2016), which can be included in the family of the signal peptide class, since it modulates skin protein turnover, but apparently through a molecular mechanism based on inhibition of collagen degradation by proteases. In fact, SA1-III is a decapeptide (Ac-Met-Gly-Lys-Val-Val-Asn-Pro-Thr-Gln-Lys-NH_2_) formally derived from the C-terminal portion of serine protease inhibitor A1 (Serpin-A1), a physiological inhibitor of neutrophil elastase, a protease involved in the degradation of ECM components.

**Figure 2:**
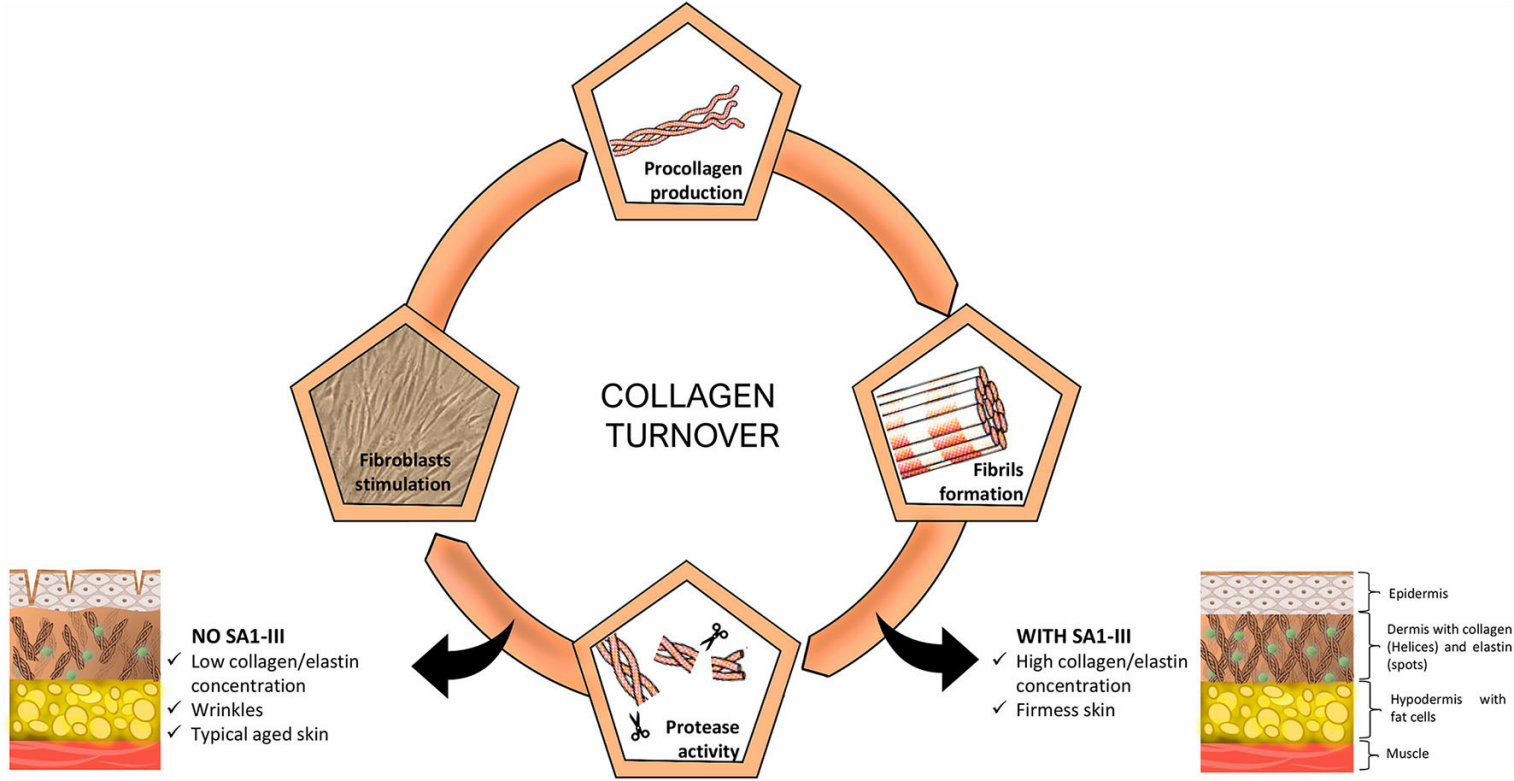
Influence of SA1-III peptide (KP1) on collagen turnover in human dermis.

Bioactivity of peptide SA1-III is summarized in Figure 2. In particular SA1-III tested in cultured human dermal fibroblasts, showed a good collagen modulation, protecting collagen against degradation without detectable actions on biosynthesis, acting at reasonably low concentrations, and non-interfering with cell proliferation (Cipriani et al., 2018).

## Sustainable Synthesis of Bioactive Peptides

Synthesis of peptides is essentially based on two relevant strategies: liquid-phase and solid-phase peptide synthesis (*LPPQ* and *SPPS*, respectively). Hybrid procedures are often convenient particularly in the case of longer sequences (Isidro-Llobet et al., 2019). The most important drawback of these approaches concerns environmental impact of most of the chemicals used in the synthetic procedures. Therefore, researchers should always be mindful in decreasing chemical pollutions generated during the laboratory operations. Considering that most of the cosmeceutical peptides are short sequences, their production by recombinant strategies is not convenient. Peptide chemists have to make an effort in developing more sustainable chemical strategies for the production of these bioactive cosmeceuticals. Indeed, research in this field is active as shown in recent papers, particularly aimed to microwave-assisted SPPS. Special attention is paid to alternative greener coupling reagents such as ethyl 2-cyano-2-(hydroxyimino)acetate, named also *oxyma*, replacing the commonly used HOBt and HOAt benzotriazole-based activators (Subirós-Funosas et al., 2009). Substitution of the hazardous solvent N,N-Dimethylformamide (DMF) by the biomass-derived γ-valerolactone was recently reported to achieve yields comparable to standard methodologies and proposed as the greenest approach, in terms of the solvent used, waste generated and energy efficiency (Kumar et al., 2018).

## Conclusions

In this mini-review we inquired the mostly used cosmeceutical peptides in the last decade. Noteworthy, cosmeceutical peptides are mainly characterized by short sequences as depicted in Figure 1. This represent a remarkable advantage in terms of production cost and scale-up.

The analysis of the recent literature on bioactive peptides as ingredients of cosmeceutical products shows that an increasing number of scientific studies support the claimed biological activity of these peptides, thus rationally underpinning the growth of this field in the framework of a sustainable wellness economy.

## Author Contributions

FE, PL, RL, PR, and AMP contributed to discuss mostly recent data reported in relevant scientific papers selected from the literature, to identify peptides as active ingredients in cosmeceutical products, and their possible biological role, in the framework of the wellness economy. All authors contributed to write the present mini-review.

## Conflict of Interest

FE was employed part-time by the company Espikem Srl. The remaining authors declare that the research was conducted in the absence of any commercial or financial relationships that could be construed as a potential conflict of interest.
